# Corneal densitometry in Chinese adults with healthy corneas: associations with sex, age, ocular metrics, and optical characteristics

**DOI:** 10.1186/s12886-024-03500-y

**Published:** 2024-05-31

**Authors:** Jiliang Ning, Siyu Sun, Qiaosi Zhang, Lin Jin, Xiaoyu Liu, Jun Xu, Lijun Zhang

**Affiliations:** Department of Ophthalmology, Liaoning Provincial Key Laboratory of Cornea and Ocular Surface Diseases, Liaoning Provincial Optometry Technology Engineering Research Center, The Third People’s Hospital of Dalian, Dalian Municipal Eye Hospital, Dalian, China

**Keywords:** Corneal densitometry, Generalized estimating equations, Sex differences, Ocular parameters, Axial length

## Abstract

**Background:**

Standardized corneal densitometry (CD) values in large samples of healthy Chinese individuals are scarce. Therefore, we aimed to determine the standard CD values using a Scheimpflug camera in healthy corneas, investigate the correlations of sex, age, and ocular parameters with corneal density, and explore the impact of corneal density on the forward scattering and optical quality of the eye.

**Methods:**

This retrospective observational study involved 990 healthy Chinese individuals, including 494 males and 496 females (mean age: 23.88 ± 6.90 years). The CD values at various depths and radial areas of 0–12 mm were measured using a Scheimpflug camera. Densitometric measurements were expressed in standardized grayscale units (GSU). The optical scatter index (OSI), modulation transfer function cutoff values (MTF_cutoff_), and Strehl’s ratio (SR) were also determined using an optical quality analysis system.

**Results:**

The average CD within a 12 mm diameter area was 16.26 ± 1.35 GSU. The highest and lowest optical densities at different depths were observed in the anterior (21.41 ± 2.16 GSU) and posterior (12.00 ± 1.01 GSU) layers, respectively (*P* < 0.001). Similarly, the maximum and minimum optical densities at different radial areas were observed in the 10–12 mm (14.09 ± 0.93 GSU) and 2–6 mm (25.93 ± 4.77 GSU) circles, respectively (*P* < 0.001). There was no significant difference in the average CD within a 12 mm diameter area between males and females (*P* > 0.05). However, upon adjusting for age, central corneal thickness (CCT), corneal curvature, white-to-white (WTW) corneal diameter, and axial length, females exhibited a greater average CD within the 12 mm diameter and in the 6–10 mm and 10–12 mm circles than males. Age-related changes in CD were evident, except in the 2–6 mm circle. CCT, corneal curvature, WTW corneal diameter, and partial depth correlated with CD in the radial area, and CD in different areas correlated with the OSI, MTF_cutoff_, and SR (*P* < 0.05).

**Conclusions:**

This study provides the normative CD measurement data of Chinese adults with healthy corneas, emphasizing the significance of sex, age, CCT, corneal curvature, and WTW corneal diameter in CD evaluation. Notably, elevated CD can lead to increased forward scattering within the eye, thereby affecting the optical quality.

## Background

The cornea, a vital refractive element of the eye, is crucial in maintaining proper vision by ensuring good transparency of the refractive medium. Corneal infections, stromal keratitis, corneal dystrophy, keratoconus, and other corneal diseases can decrease corneal transparency and increase light scattering [1]. Scheimpflug photography is an innovative imaging technique that measures backward-scattered corneal light and quantifies corneal transparency. The Pentacam HR (Oculus, Wetzlar, Germany) is an innovative anterior segment analysis instrument based on the Scheimpflug photography principle. This instrument efficiently captures corneal backscattered light within a 12 mm diameter range and analyzes the average corneal densitometry (CD) value at various depths and diameters around the corneal apex [2]. Previous studies have extensively investigated CD distribution in healthy individuals. However, standardized CD values in large samples of healthy Chinese individuals are scarce [3–5]. To our knowledge, this study is currently the largest measurement of CD in a healthy Chinese population.

In this study, we aimed to establish standardized CD values for Chinese individuals, examine the influence of sex, age, corneal keratometry, central corneal thickness (CCT), white-to-white (WTW) corneal diameter, and axial length on CD, and investigate the impact of CD on forward light scattering and the optical quality of the eye.

## Methods

### Study participants

This retrospective observational study was conducted at the Ophthalmology Department of Dalian Third People’s Hospital. In total, 990 healthy individuals who underwent eye examinations between December 2020 and May 2022 were included in the study. Based on the pre-experiment findings, no significant difference in optical density was observed between the patients’ two eyes. However, to avoid any potential bias due to the correlation between the eyes, only the participants’ right eyes were analyzed in this study. Before participation, all individuals refrained from wearing contact lenses for at least 2 weeks and showed no signs of corneal opacity upon slit lamp examination. Individuals with eye diseases beyond refractive error (such as keratoconus, suspected keratoconus, other types of corneal ectasia, active eye disease, and dry eye disease) and those with systemic diseases that may affect the eye (such as diabetes, immune diseases, or systemic infectious diseases) were excluded from the study. The study protocol was approved by the Ethics Committee of Dalian Third People’s Hospital (Approval Number: 2022-039-001). The research was conducted following the Declaration of Helsinki, and all participants provided informed consent.

### Measurement technique

**Fig. 1:**
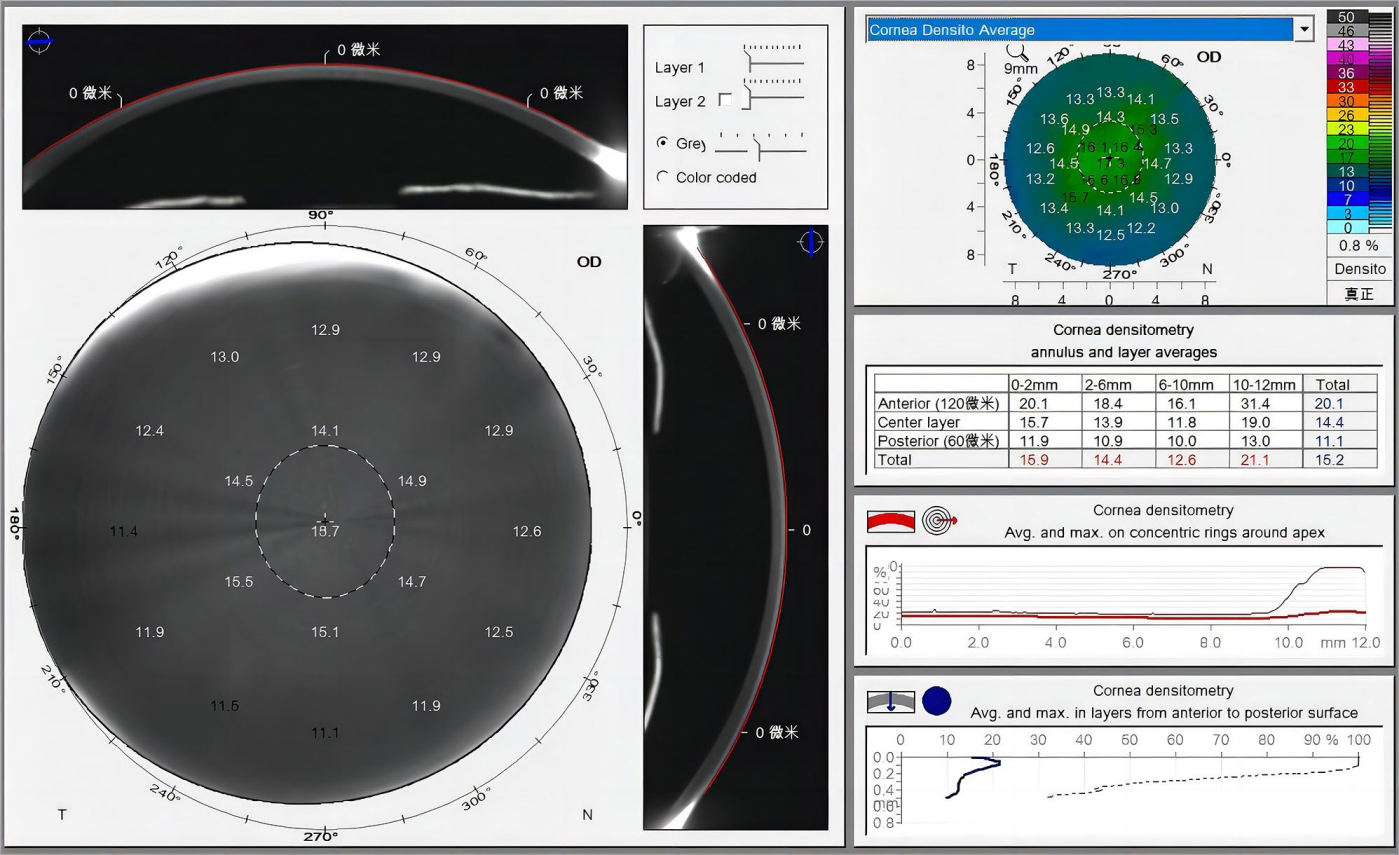
Screen data output of the Scheimpflug corneal densitometry assessment

The same physician performed all examinations. Corneal transparency was evaluated using the Pentacam HR densitometry software. During the examination, the participants were placed in a windowless dark room. The participant’s chin was placed on a chin rest, and the forehead was positioned against the forehead band. The participants’ eyes were kept horizontal, and their heads were covered with a cloth that shielded light. To acquire measurements, the device automatically captured 25 corneal Scheimpflug images of different meridians. The results were saved only if the acquisition quality specification value was acceptable and there were no apparent obstructions from the eyelids or eyelashes. The Pentacam internal CD analysis software examined the backscattered light within a 12 mm diameter range, as depicted in Fig. 1. Densitometry values are expressed in grayscale units (GSU), ranging from 0 to 100. A GSU of zero indicates minimal light scattering, denoting that the cornea is completely transparent. However, a GSU of 100 indicates maximal light scattering, denoting that the cornea is completely opaque. CD measurements were taken in four concentric radial areas (0–2 mm, 2–6 mm, 6–10 mm, and 10–12 mm) centered on the corneal apex. The anterior layer (front 120 μm), posterior layer (back 60 μm), and the central layer between the anterior and posterior layers were also analyzed. The Pentacam HR was used to measure the anterior segment parameters, including corneal keratometry, CCT, and WTW corneal diameter. Additionally, axial length was measured using the IOL Master 500 (Carl Zeiss Meditec, Germany), and the optical quality analysis system (OQAS™II, Visiometrics, Spain) was used to determine the ocular optical scatter index (OSI), modulation transfer function cutoff values (MTF_cutoff_), and Strehl’s ratio (SR).

### Statistical analysis

Statistical analysis was conducted using IBM SPSS Statistics for Windows, version 26.0. The measurement data were assessed for normal distribution using the Shapiro-Wilk test. Normally distributed data were presented as mean ± standard deviation with a 95% confidence interval. Repeated measures analysis of variance was used to compare the average CD measurements in different areas. The Bonferroni test was employed as a post-hoc test for multiple group comparisons. An independent samples t-test was conducted to compare the CD differences between sexes. Generalized estimating equations (GEEs) were used to investigate the effects of sex, age, CCT, corneal curvature, WTW corneal diameter, and axial length on CD. The results of this model are reported using unstandardized beta coefficients and standard errors. Pearson correlation analysis was used to investigate the correlation between the OSI, MTF_cutoff_, SR, and CD. Statistical significance was set at *P* < 0.05.

## Results

This study involved 990 healthy Chinese individuals with an average age of 23.88 ± 6.90 years. The participants included 494 males and 496 females, with analyses focusing solely on their right eyes. The average axial length of the participants’ eyes was 25.80 ± 1.10 mm. Table 1 presents the anterior segment parameters measured using Pentacam HR, with OSI, MTF_cutoff_, and SR values determined using OQAS™II.

**Table 1:** Demographic data and ocular parameters of the subjects

Parameter	Mean ± SD	95% CI
Number of eyes	990	
Sex(male/female)	494/496	
Age, years	23.88 ± 6.90	23.45–24.32
CCT, µm	551.76 ± 30.46	549.86-553.66
Corneal keratometry, D	43.36 ± 1.40	43.28–43.45
WTW, mm	11.80 ± 0.41	11.77–11.82
Axial length, mm	25.80 ± 1.10	25.73–25.87
OSI	0.68 ± 0.46	0.64–0.72
MTF_cutoff,_ cpd	39.90 ± 10.10	39.01–40.79
SR	0.23 ± 0.06	0.22–0.23

CCT, central corneal thickness; WTW, white-to-white corneal diameter; OSI, optical scatter index; MTF_cutoff_, modulation transfer function cutoff values; SR, Strehl’s ratio

**Fig. 2:**
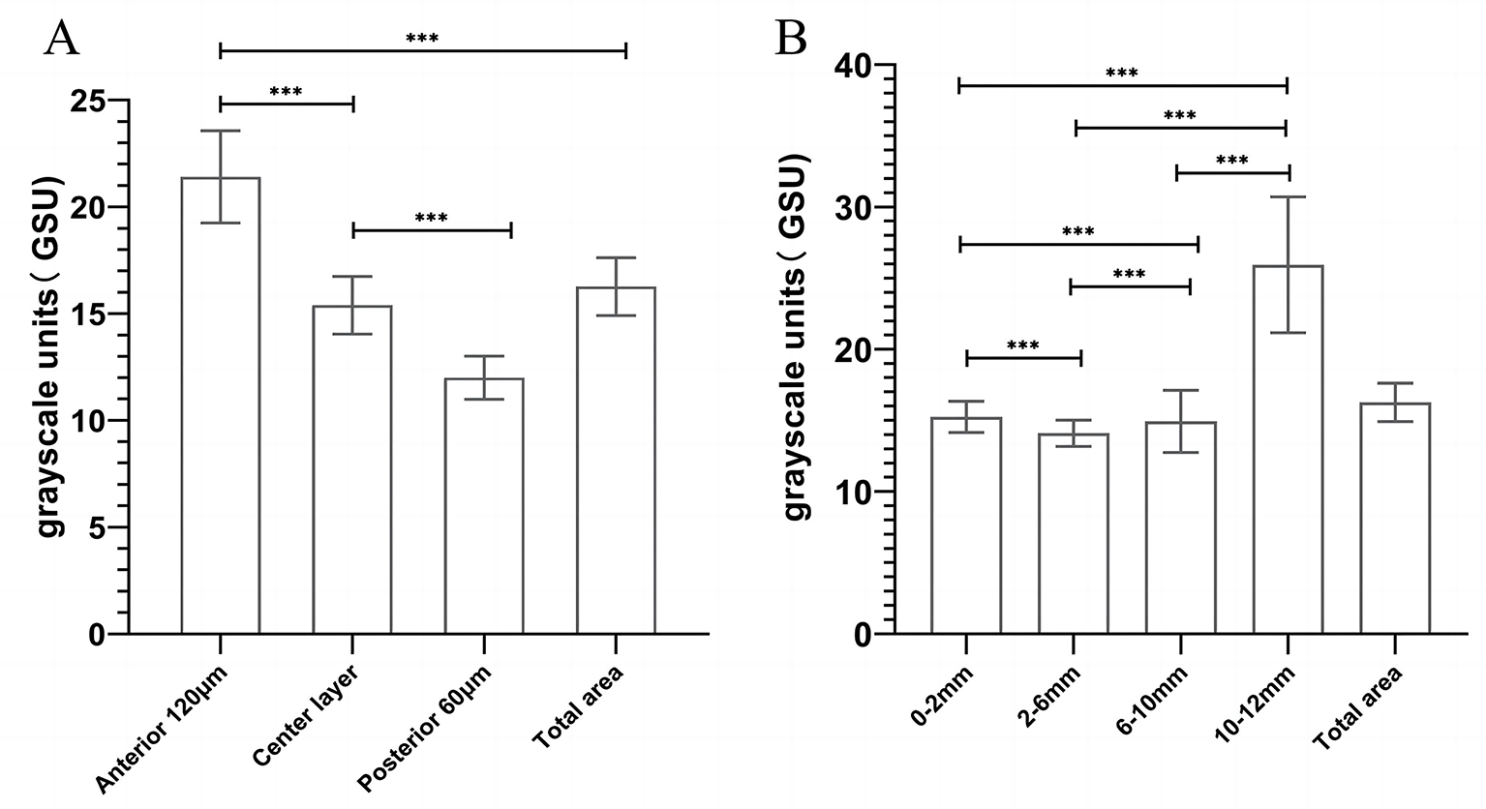
CD measurements in different radial areas and depths. (**A**) CD subdivided based on different radial areas. (**B**) CD subdivided based on different corneal layers. *** refers to a statistical significance of *P* < 0.001

Table 2 and Fig. 2 present the CD values for different regions. The mean CD within the 12 mm diameter range was 16.26 ± 1.35 GSU. The CD of the four concentric radial areas showed statistically significant differences when divided based on the radial area. Among them, the 2–6 mm circle exhibited the lowest CD of 14.09 ± 0.93 GSU, whereas the 10–12 mm circle exhibited the highest CD of 25.93 ± 4.77 GSU (*P* < 0.001). Statistical significant differences in CD across various depths were observed; specifically, the anterior layer recorded the highest CD at 21.41 ± 2.16 GSU, whereas the posterior layer exhibited the lowest at 12.00 ± 1.01 GSU (*P* < 0.001).

**Table 2:** Mean and SD of corneal densitometry in different corneal radial areas and depths in healthy individuals

	Zones	Mean ± SD	95% CI
Anterior 120 μm	0–2 mm	19.75 ± 1.68	19.65–19.86
	2–6 mm	18.47 ± 1.47	18.37–18.55
	6–10 mm	19.54 ± 3.23	19.33–19.74
	10–12 mm	35.01 ± 8.44	34.48–34.53
	0–12 mm	21.41 ± 2.16	21.27–21.54
Center layer	0–2 mm	14.60 ± 1.06	14.53–14.66
	2–6 mm	13.24 ± 0.87	13.19–13.29
	6–10 mm	13.92 ± 2.14	13.79–14.05
	10–12 mm	25.05 ± 4.68	24.75–25.34
	0–12 mm	15.39 ± 1.35	15.31–15.47
Posterior 60 μm	0–2 mm	11.38 ± 0.76	11.33–11.42
	2–6 mm	10.76 ± 4.26	10.49–11.02
	6–10 mm	11.34 ± 1.44	11.25–11.43
	10–12 mm	17.78 ± 3.50	17.56–17.99
	0–12 mm	12.00 ± 1.01	11.93–12.06
Total thickness	0–2 mm	15.25 ± 1.09	15.18–15.31
	2–6 mm	14.09 ± 0.93	14.04–14.15
	6–10 mm	14.93 ± 2.19	14.79–15.06
	10–12 mm	25.93 ± 4.77	25.64–26.23
	0–12 mm	16.26 ± 1.35	16.18–16.35

Table 3 compares the CD differences between the sexes. The average CD within the 12 mm diameter range was 16.19 ± 1.33 GSU in males and 16.33 ± 1.37 GSU in females. The two groups showed no statistically significant differences (*P* = 0.11). Female patients exhibited a higher CD in the anterior, middle, posterior, and overall layers within the 6–10 mm zone and in the middle, posterior, and overall layers within the 10–12 mm zone (*P* < 0.05). Moreover, they had denser corneas in the middle and posterior layers of the 12 mm diameter range (*P* < 0.05). However, males displayed higher CD in the anterior, middle, and overall layers in the 0–2 mm zones and the anterior layer within the 2–6 mm zone compared with females (*P* < 0.01).

**Table 3:** Sex differences in corneal densitometry at various radial areas and depths in healthy individuals

	Zones	Male		Female			
		Mean ± SD	95% CI	Mean ± SD	95% CI	t	*P*
Anterior 120 μm	0–2 mm	20.04 ± 1.63	19.89–20.18	19.47 ± 1.69	19.32–19.62	5.409	0.000
	2–6 mm	18.63 ± 1.41	18.51–18.75	18.30 ± 1.50	18.17–18.44	3.536	0.000
	6–10 mm	19.31 ± 3.20	19.03–19.59	19.76 ± 3.25	19.47–20.05	-2.187	0.029
	10–12 mm	35.29 ± 8.89	34.51–36.08	34.72 ± 7.96	34.02–35.42	1.082	0.280
	0–12 mm	21.53 ± 2.21	21.33–21.72	21.28 ± 20.10	21.10-21.47	1.784	0.075
Center layer	0–2 mm	14.69 ± 1.02	14.60-14.78	14.50 ± 1.09	14.41–14.60	2.810	0.005
	2–6 mm	13.26 ± 0.83	13.18–13.33	13.22 ± 0.90	13.14–13.30	0.613	0.540
	6–10 mm	13.57 ± 2.04	13.39–13.75	14.27 ± 2.18	14.07–14.46	-5.207	0.000
	10–12 mm	24.44 ± 4.76	24.02–24.86	25.65 ± 4.52	25.26–26.05	-4.121	0.000
	0–12 mm	15.24 ± 1.26	15.13–15.35	15.54 ± 1.42	15.42–15.67	-3.595	0.000
Posterior 60 μm	0–2 mm	11.38 ± 0.74	11.31–11.45	11.37 ± 0.77	11.31–11.44	0.200	0.842
	2–6 mm	10.72 ± 4.09	10.36–11.08	10.80 ± 4.43	10.41–11.19	-0.292	0.770
	6–10 mm	11.02 ± 1.32	10.90-11.14	11.67 ± 1.49	11.54–11.80	-7.251	0.000
	10–12 mm	17.09 ± 3.48	16.79–17.40	18.45 ± 3.38	18.15–18.75	-6.246	0.000
	0–12 mm	11.80 ± 0.92	11.71–11.88	12.20 ± 1.06	12.11–12.29	-6.400	0.000
Total thickness	0–2 mm	15.37 ± 1.06	15.28–15.47	15.12 ± 1.11	15.02–15.21	3.767	0.000
	2–6 mm	14.14 ± 0.89	14.06–14.22	14.04 ± 0.96	13.96–14.13	1.670	0.095
	6–10 mm	14.63 ± 2.10	14.44–14.81	15.23 ± 2.23	15.03–15.42	-4.368	0.000
	10–12 mm	25.60 ± 5.00	25.15–26.03	26.27 ± 4.52	25.87–26.67	-2.227	0.026
	0–12 mm	16.19 ± 1.33	16.08–16.31	16.33 ± 1.37	16.21–16.45	-1.618	0.106

GEEs were used to evaluate the relationship between CD in different concentric radial and depth areas and factors, including sex, age, CCT, corneal curvature, WTW corneal diameter, and axial length (Table 4). Females also exhibited a higher CD than males across different radial areas and depths besides the CD of the posterior layer (*P* < 0.05). Age showed no correlation with the cornea’s CD in the 2–6 mm circle; however, a negative correlation was observed with CD in the 0–2 mm circle (*P* < 0.05). Conversely, age was positively associated with CD across other radial areas and depths (all *P* < 0.05). CCT demonstrated a correlation with CD in the 0–2 mm and 2–6 mm circles and the anterior layer (*P* < 0.01). The corneal curvature and the 6–10 mm circle were negatively associated with the CD in the anterior, middle, and total layers (*P* < 0.01). The WTW corneal diameter was positively correlated with CD in the 0–2 mm and 2–6 mm circles but negatively correlated with CD in other radial areas and the anterior, middle, and posterior layers. Furthermore, a negative correlation was observed between axial length and CD in a 10–12 mm circle (*P* < 0.01).

**Table 4:** Generalized estimating equations were employed to assess the association between corneal densitometry and sex, age, CCT, corneal curvature, WTW corneal diameter, and axial length

Parameter		0–2 mm	2–6 mm	6–10 mm	10–12 mm	Anterior	Center	Posterior	Total
Sex	*β*	-0.106	-0.41	-0.375	-1.116	-0.745	-0.164	0.003	-0.317
	*SE*	0.074	0.065	0.119	0.281	0.138	0.084	0.060	0.083
	*P*	0.153	0.527	0.002	< 0.001	< 0.001	0.050	0.964	< 0.001
Age, year	*β*	-0.016	0.002	0.107	0.076	0.052	0.056	0.051	0.053
	*SE*	0.005	0.005	0.010	0.020	0.011	0.007	0.005	0.007
	*P*	0.003	0.688	< 0.001	< 0.001	< 0.001	< 0.001	< 0.001	< 0.001
CCT,µm	*β*	0.003	0.004	0.003	0.005	0.007	-0.001	-0.001	0.002
	*SE*	0.001	0.001	0.002	0.004	0.002	0.001	0.001	0.001
	*P*	0.007	< 0.001	0.092	0.183	< 0.001	0.605	0.454	0.076
Corneal keratometry, D	*β*	0.058	0.002	-0.181	0.001	-0.153	-0.112	-0.004	-0.093
	*SE*	0.031	0.027	0.050	0.105	0.056	0.033	0.026	0.034
	*P*	0.064	0.955	< 0.001	0.990	0.006	0.001	0.870	0.007
WTW, mm	*β*	0.503	0.203	-2.942	-7.463	-2.052	-1.413	-0.798	-1.436
	*SE*	0.104	0,090	0.165	0.367	0.192	0.103	0.079	0.109
	*P*	< 0.001	0.023	< 0.001	< 0.001	< 0.001	< 0.001	< 0.001	< 0.001
Axial length, mm	*β*	-0.025	-0.022	-0.071	0.832	0.113	0.036	-0.023	0.043
	*SE*	0.034	0.029	0.060	0.136	0.065	0.037	0.027	0.039
	*P*	0.451	0.457	0.236	< 0.001	0.085	0.329	0.393	0.270

CCT, central corneal thickness; WTW, white-to-white corneal diameter; *β*, unstandardized beta co-efficient; *SE*, standard error

Table 5 presents the correlation between CD across various radial areas and the eye’s forward scattering and optical quality. CD measurements within the 0–2 mm, 2–6 mm, and 10–12 mm circles, and the average CD of the 12 mm diameter range positively correlated with OSI (*P* < 0.05). Conversely, CD values for circles ranging from 2 to 6 mm, 6–10 mm, and 10–12 mm, and the average CD within the 12 mm diameter range, demonstrated a negative association with the MTF_cutoff_ (*P* < 0.05). Furthermore, CD across distinct radial areas and the average CD of the 12 mm diameter displayed a negative correlation with SR (*P* < 0.05).

**Table 5:** The correlation between corneal densitometry at different radial areas and forward scattering and optical quality

Parameters		0–2 mm	2–6 mm	6–10 mm	10–12 mm	0–12 mm
OSI	*r*	0.159	0.158	0.066	0.111	0.125
	*P*	0.000	0.000	0.142	0.013	0.005
MTF_cutoff_,cpd	*r*	-0.086	-0.101	-0.092	-0.128	-0.130
	*P*	0.054	0.024	0.041	0.004	0.001
SR	*r*	-0.096	-0.113	-0.114	-0.148	-0.149
	*P*	0.033	0.011	0.011	0.001	0.001

OSI, optical scatter index; MTF_cutoff_, modulation transfer function cutoff values; SR, Strehl’s ratio; *r*, Pearson correlation coefficient

## Discussion

This study presents the largest CD measurement in normal Chinese individuals. It provides information on the normal CD values of 990 eyes of 990 healthy individuals aged 16–52 years. The study also explored the correlation between CD and various factors, such as sex, age, and ocular parameters, including CCT, corneal curvature, WTW corneal diameter, and axial length. The average CD value measured in this study was 16.26 ± 1.35 GSU. Previous studies reported normal CD values in various regions and age groups. Ni Dhubhghaill et al. reported an average CD value of 17.94 ± 3.89 GSU among 445 healthy white individuals aged 20.2 and 84.2 years [2].

Similarly, Garzón et al. investigated 338 healthy Spanish individuals aged 20–51 years and reported a mean CD of 16.46 ± 1.85 GSU [6]. Pakbin et al. observed a mean CD value of 14.86 ± 2.37 in 261 photorefractive keratectomy candidates aged 21–40 years [7]. The variation in the results could be attributed to differences in participants’ geographical location and age range. The light intensity in the environment could also have influenced the findings. Bahar et al. found that densitometry values tended to increase with increasing levels of ambient light [8]. Therefore, our study maintained a controlled dark room setting and utilized a black shading cloth to shield the patient’s head to mitigate this potential influence, ensuring a consistent environment for CD value measurements.

Based on our findings, the anterior 120 μm exhibits the highest CD, whereas the posterior 60 μm exhibits the lowest CD. Notably, this contrasts with the confocal microscopy results, which identified maximum backscattering in the posterior corneal layer [9]. Confocal microscopy utilizes a contact gel to minimize reflections at the air-corneal interface, which can cause measurement errors due to corneal deformation due to simultaneous contact. Additionally, the two techniques have different imaging principles. Scheimpflug analysis involves examining the cornea at ± 45º, whereas confocal microscopy employs vertical imaging, which may result in increased specular reflected light. The differences in the corneal optical density at different levels can be attributed to the anisotropy of the corneal microstructure. Specifically, the anterior corneal layer houses numerous corneal cell nuclei, resulting in increased backscatter compared with the middle and posterior layers [10]. Unlike the orderly structured middle and posterior corneal stroma, the anterior stroma has a denser collagen arrangement, heightened interfiber cross-linking, and a richer arcuate spring structure. These characteristics contributed to the higher CD [11]. The density measurement values in the different radial areas showed the lowest and highest values in the 2–6 mm and 10–12 mm circles, respectively. This finding is consistent with that of Garzón et al. [4, 6]. The increased retroreflection in the peripheral region may be attributed to the circumferential arrangement of the peripheral corneal collagen fibers relative to the limbus and the relatively large diameter of the elastic fibers [12, 13]. The variability of corneal diameter in WTW may impact CD in the 10–12 mm circle. Specifically, when the corneal diameter falls below 12 mm, the density measurement analysis might encompass the limbal portion of the cornea, which has a larger CD.

The impact of sex on CD remains unclear, as there is no consensus among studies. Notably, some studies have found no significant association between sex and CD [2, 4]. However, Hillenaar et al. used confocal microscopy to quantify CD values, revealing that males exhibited a 3.5% higher CD value than females [9]. Our investigation showed that CD was greater in males within the cornea’s central region, whereas females exhibited a larger CD than males in the peripheral area. When the generalized linear estimating equation was applied to account for the influence of age, CCT, corneal curvature, WTW corneal diameter, and axial length, the CD was higher in females than in males. This finding aligns with a previous study by Garzón et al. [6]. The reasons for these sex differences in CD measurements are currently unknown. Therefore, further research is required to explore the possible effects of sex hormones on corneal transparency.

Our study revealed a positive correlation between the CD value of each layer and the average CD value in the 12 mm corneal diameter area with age. This suggests increased diameter and intermolecular cross-linking of collagen fibrils in the corneal stroma with age, leading to increased corneal backscatter levels [14]. The corneal endothelial cells are pivotal in preserving transparency. The density of these cells and the proportion of hexagonal cells are directly associated with CD [15, 16]. Consequently, age-related alterations in the endothelial layer could contribute to the observed increase in CD values. Consistent with the study conducted by Asrar and Ni Dhubhghaill et al. [2, 17], our findings indicated a strong association between peripheral corneal backscatter and age. This correlation may be attributed to the development of age-related limbal deformations, including the farinata, Arcus senilus/lipoides, crocodile shagreen, Vog’s white limbal girdle, and Hassal-Henle bodies. Notably, we observed a negative correlation between the 0–2 mm backscatter and age, necessitating further investigation of its underlying causes.

Previous reports have consistently indicated no correlation among CD measurements, corneal curvature, and CCT [4, 6]. However, in our study, we observed a positive correlation between the central 0–2 mm and 2–6 mm circles and the CD value of the anterior layer with CCT. In contrast, a negative correlation was observed between the 6–10 mm circle and the average CD value within the 12 mm diameter range concerning the refractive power of the anterior corneal surface. A negative correlation was also identified between the corneal WTW diameter and the average and peripheral CD values. However, there was a positive correlation between corneal WTW diameter and the CD in the central area. This suggests that corneas of different diameters may have variations in collagen fibrils or stromal tissue, leading to differences in light backscattering characteristics. In a study by Garzón et al., no correlation was observed between the spherical equivalent power and the CD across different corneal areas [6]. This finding suggests that the degree of myopia does not affect corneal transparency. Notably, our study highlighted a positive correlation between CD in the 10–12 mm circle and axial length. However, the optical density in this area should be interpreted cautiously because it is located near the limbus.

The light scattered by the human eye can be classified into forward and backscattering based on its direction. Forward scattering can decrease the clarity of a retinal image and affect the perception of contrast [18]. Backscattering can reduce the total amount of light reaching the retina and selectively filter light at different angles of incidence, potentially affecting vision under low-light conditions. The relationship between forward and backward reflections becomes intricate under conditions of low scattering [19]. Wu et al. used C-quant to assess healthy eyes and discovered a significant correlation between CD and ocular stray light [20]. Patel et al. explored corneal backscatter post-penetrating keratoplasty and observed a gradual increase in backscattering over time [18]. This increase correlated with a decline in high- and low-contrast visual acuity.

The optical quality analysis system uses dual channels to capture images of point-light sources on the retina. This mechanism facilitates the analysis of the point spread function, subsequently yielding metrics such as OSI, MTF_cutoff_, and SR [21, 22]. The OSI values represent the ratio of peripheral light intensity to the central peak light intensity in the retinal image. It measures the ratio of the light intensity in the ring area between 12 and 20 arcmin to that in 1 arcmin. The OSI provides information on the human eye’s level of forward scattering. MTF_cutoff_ represents the spatial frequency at which the modulation transfer function reaches 0.01, indicating an extreme resolution. The SR reflects the light intensity ratio between the actual and ideal optical systems. A higher value of MTF_cutoff_ and SR indicates better optical quality. To our knowledge, this is the first study to investigate the correlation between CD and OSI and optical quality in healthy individuals. Our findings indicate a positive correlation between CD and OSI and a negative correlation between the MTF_cutoff_ and SR. These results highlight the influence of corneal backscattering on forward scattering and the overall optical quality of the human eye.

This study has some limitations. First, it focused on young and middle-aged healthy individuals, restricting the comprehensive evaluation of corneal optical density and age. Second, China is a vast country, and this study mainly focused on the individuals who underwent refractive examinations in this medical institution, which may not represent the corneal optical density levels of the entire country. Future studies should include a wider age range and more regions.

## Conclusions

To date, this study is the largest investigation of densitometric measurements of transparent corneas in healthy Chinese individuals. It presents the normative values of corneal density in various regions and investigates the impact of factors such as sex, age, axial length, and corneal parameters on corneal density. Additionally, we established a correlation between corneal density and forward scattering and the optical quality of the human eye.

## Data Availability

The raw data supporting the conclusions of this article will be made available by the authors, without undue reservation.

## Abbreviations

CD: Corneal densitometry
GSU: Standardized grayscale units
OSI: Optical scatter index (OSI)
MTF_cutoff_: Modulation transfer function cutoff values
SR: Strehl’s ratio
CCT: Central corneal thickness
WTW: White-to-white
GEE: Generalized estimating equations
OQAS™II: Optical quality analysis system
